# Emotional responses to mortality salience: Behavioral and ERPs evidence

**DOI:** 10.1371/journal.pone.0248699

**Published:** 2021-03-17

**Authors:** Shiyun Huang, Hongfei Du, Chen Qu

**Affiliations:** 1 Center for Studies of Psychological Application, South China Normal University, Guangzhou, China; 2 Guangdong Key Laboratory of Mental Health and Cognitive Science, South China Normal University, Guangzhou, China; 3 Department of Psychology, Guangzhou University, Guangzhou, China; Centre National de la Recherche Scientifique, FRANCE

## Abstract

Terror Management Theory (TMT) suggests that death-related thoughts activate proximal defense which allows people to suppress or rationalize death awareness. So far there is no direct evidence to support the emotional response in the proximal defense process. The current research aimed to address this issue by examining behavioral (e.g., accuracy and reaction time) and neural responses (e.g., P1 and N400 amplitude) related to emotional arousal following death-related thoughts during proximal defense. Before engaged in emotional words (e.g., anxiety, fear and neutral) judgment task, participants answered questions that referred to emotional and physical changes about death to induce mortality salience (MS). In the control condition, participants received similar instructions concerning the experience of watching TV. Behavioral results showed that longer reaction time of words was seen in control group than MS group. The ERPs results showed that after reminders of death-related thoughts, in condition of MS, fear words elicited larger P1 ERP amplitudes, while the control group did not have this effect, which might reflect that emotional words caused different early attention patterns between MS group and control group. Moreover, compared with control group, larger N400 ERP amplitudes were elicited in condition of MS, suggesting larger cognitive inhibition of words processing caused by emotional reaction. The above results indicate that the early stages after mortality salience will induce fear and anxiety, but soon these negative emotions are suppressed and are at a lower level of accessibility. This result provides electrophysiological evidence for the proximal defense hypothesis of terror management theory.

## Introduction

All the people have limited lifetime and have to face inevitable death. In general, the inevitability of death does not lead one to live cautiously or have a more negative impact in life. Terror Management Theory is one of the most influential theories related to death in recent years, it provides a theoretical basis for people to understand the impact of death information on human behavior motivation and psychology [1]. Terror management theory believes that some psychological defense mechanisms help us alleviate the fear and anxiety caused by death-related thoughts. The awareness of mortality engenders individuals to seek for potential buffers, such as eating healthy food and performing daily exercise. Beyond physical efforts to reduce death concerns, human beings have developed and relied on psychological buffers against fear about death [2, 3]. After mortality salience, the thoughts that death is inevitable will affects people’s emotion, cognition and behavior [4]. Management of thought of death and its subsequent reactions has been held to be universal. The current study aims to answer whether emotional responses are involved and what emotions (e.g., anxiety or fear) can be aroused in terror management process by providing behavioral and neural evidence.

### Terror management and dual-process model

Terror management theory posits a dual-process model, proximal defense and distal defense in response to death-related thoughts [5]. When thoughts of death are induced, conscious concerns about death are first experienced. What we utilize to fight against conscious concerns is proximal defense. During proximal defense, rational efforts are engaged, for instance, individuals tend to suppress death-related thoughts by focusing on death-unrelated activities or deny death by thinking that death is far away because of their young age and excellent health [6].

Distal defense, the second process of defense, emerges when concerns on death are salient in unconsciousness. Although death-related thoughts are not salient in focal attention in this process, unconscious terror is still accessible and exert effects in an implicit way [7, 8]. Suppression and denial are not useful anymore in distal defense because of the unconscious nature of terror. Instead of suppression and denial, distal defense mitigates terror of death through symbolic assurance of a meaningful, valuable, and enduring life. Cultural worldviews and self-esteem are two symbolic buffers of distal defense. With defense in cultural worldviews (e.g., identify more with the worldview of one’s culture) and maintenance of self-esteem (e.g., enhance self-esteem), one can alleviate unconscious terror of death.

### Terror management and emotions

Terror management theorists propose that both proximal and distal defenses are cognitive without emotional involvement, and call it affect-free hypothesis [3, 9]. Even if there are sometimes subjective experience of emotions, emotions would not explain the effects of death-related thoughts on cultural worldview and self-esteem defense [6, 10, 11].

However, this affect-free view has been challenged by recent research [12]. Routledge and his colleagues found that MS manipulation increased self-reported anxiety, though only among people who lack perceptions of meaning in life [13]. Moreover, people with low self-esteem showed increased anxiety during distal defense when thoughts of death were outside of focal attention (i.e., assessing anxiety after a delay following MS) [14]. And a negative affect respond to thoughts of death was found on an implicit level [15].

Second, fear has also been identified as an emotional reaction in response to MS. Lambert and his colleagues [16, 17] suggested that previous hundreds of studies in the terror management literature did not show the effects of salient thoughts of death on emotions, because these studies mostly assessed positive and negative affect in general, that may not be directly related to thoughts of death. Instead, the study of Lambert [17] focused on the effects of MS on fear and anxiety, the two types of emotions possibly induced by thoughts of death, rather than other general negative affect. This study showed activation of fear and terror-related sentiments in condition of MS, but MS did not show effects on anxiety that was in contrast to Routledge’s findings which anxiety was occurred after MS [13, 14]. It is worth to note that Lambert assessed emotional reactions at the proximal defense stage [17], indicating that emotions might be involved in not only proximal defense, but also distal defense.

Third, other specific types of emotions were also found in recent terror management research. Individual differences in disgust predicted worldview defense in response to MS [18]. Furthermore, Webber [19] suggested that only when disgust was present could threats to cultural worldviews induce unconscious death concerns assessed by death thought accessibility. In addition, DeWall [20] found feelings of joy following MS.

Taken together, recent research seemed to suggest that emotional components are involved in terror management processes. However, there is no direct evidence to support the emotional response in the proximal defense process and what types of emotions (e.g., anxiety or fear) can be aroused in terror management process are still unclear. The present study aims to uncover the types of emotions (anxiety and fear) along with thoughts of death during the proximal defense process by utilizing ERPs.

### Terror management and ERPs

The purpose of this research is to solve a core issue in terror management theory: what emotional components are involved in terror management proximal defense process? Specifically, we are interested in revealing whether anxiety and fear would be shown following thoughts of death. To answer these questions, we adopted the commonly used MS paradigm and examined whether and how MS influenced subsequent emotional reactions. Three types of words (anxiety, fear and neutral) were used as probes. After MS, participants were instructed to judge whether the words are emotional or not. Reaction time (RT) of judging words and ERPs were collected. As for ERPs, we focus to P1 and N400 components.

Early cortical response P1 has been found to reflect early visual processing, interpreting the rapidly captured neural effect to attended stimuli [21] and emotional words [22] in sensory processing. Using an emotional Stroop task [23] and dual-target rapid serial visual presentation task [24], P1 was found to be more positive for negative words compared to neutral words. Researchers suggest that P1 is associated with involuntary attention for visual verbal stimuli [25]. If emotional reactions are not induced or are inhibited in response to MS, we could expect MS group would show the same pattern of P1 elicited by emotional words with control group. On contrary, a different pattern might provide new evidence for the existing emotional response.

N400 is a component distinctly sensitive to the semantic context of stimuli [26], reflecting the difficulty on integrating words [27]. Accumulated evidence from various research in the field of emotion suggest that N400 is larger for neutral words than for negative words [28–31] and associated with semantic integration, indicating the facilitated processing of emotional words during semantic processing [27, 32, 33]. When death-related thoughts are primed, anxiety and fear might be diluted and suppressed by proximal defense and individuals would avoid confronting to such negative emotions, which would result in a more difficult integration to fear and anxiety words than that for control group. Therefore, we expect a larger N400 responding to fear and anxiety words in MS group than that in non-MS control group.

In summary, this study utilizes the MS paradigm and emotional word judgment task to investigate whether death consciousness causes emotional reactions through the behavioral responses and ERPs of the MS group and the control group under different vocabulary conditions (anxiety, fear, and neutral).

## Materials and methods

### Participants

A total of 61 healthy graduate students (39 females and 22 males; *M* = 20.49 years, *SD* = 1.69) were recruited from South China Normal University, China. Ages between experimental group and control group did not differ from each other (*t* = 1.42, *p* = .16, *1-β* = .95, *d* = .36), therefore age was not considered further here. All participants were right-handed (mean laterality = .90 attesting by the Edinburgh Handedness questionnaire [34], reporting normal vision or corrected normal visual acuity. None of the participants had a history of mental disorders, drug abuse or neurological disorders. Participants were briefed on the procedure and the possible influence to the human body. Participants read and signed informed consent prior to the experiment and had a right to quit during the experiment without any cost at any time. All methods in this study were performed in accordance with the relevant guidelines, which were declared and approved by the Human Research Ethics Committee for Non-Clinical Faculties in South China Normal University. After finishing the experiment, participants were given compensation (30 RMB per hour, equal to 4.2 USD).

### Materials stimuli

Fifty-one Chinese words were used as the stimuli in the ERP process. We translated the words used in the study of Lambert [17] into Chinese, based on which we retrieved additional words using the Chinese thesaurus. The fifty-one words were divided into three categories: fear-related words (e.g., afraid, scared, frightened), anxiety-related words (e.g., anxious, nervous, worrying), and neutral words (e.g., simple, dry, crowded). Each category comprised 17 words. We conducted a pilot study to evaluate the arousal and valence levels of words on a 7-point Likert scale. Twenty participants were asked to rate the arousal (i.e., How emotionally arousing is this word?) and valence (i.e., How negative/positive is this word?) levels of fear-related, anxiety-related, and neutral words. Analyses showed that the word categories were not significantly different in terms of arousal (*F* (2, 38) = .40, *p* = .67, *η*^*2*^_*p*_ = .02). However, the word categories significantly differed with respect to valence (*F* (2, 38) = 6.05, *p* = .005, *η*^*2*^_*p*_ = .24). Both fear-related and anxiety-related words were rated as more negative than neutral words (all *ps* < .03), but no significant difference in ratings between fear-related and anxiety-rated words (*p* = .70). The non-significant difference between fear-related and anxiety-related words satisfy our purpose of examining the potential effects of MS on them, that is, differentiate effects of MS on reactions to fear-related vs. anxiety-related word would not be accounted by the levels of arousal and valence. Considering that the word frequency and stroke may affect ERPs [35–37], we also examined number of strokes of the words and lexical frequency that were retrieved from Baidu database. Analyses showed that the words in three categories had no significant differences in number of strokes (*p* = .11) and lexical frequency (*p* = .65).

### Priming manipulation

To evoke death-related thoughts, the paradigm of mortality salience (MS) is commonly used, by which people are primed with death-related stimuli (e.g., think about the emotional and physical changes about death, exposure to imagery or words related to death). MS manipulation in this experiment was consistent with the task used by previous terror management researchers [38]. Participants were randomly assigned to the MS condition (MS group) or the control condition. In the MS condition, participants were required to answer two questions: "Describe the emotions that the thought of your own death arouses in you" and "describe what you think will happen to you when you physically die and once you are dead ". In the control condition (control group) participants were requested to fill in the daily activities that "describe the emotions when you are watching TV" and "describe what you think happens to you when you watch TV".

### Procedure

People tend to be taboo about the topic of death, or even keep silent. Therefore, before the priming manipulation, participants were told that the aim of the experiment was to investigate personality once they arrived in the lab. Prior to the tasks, participants were arranged to sit in a comfortable armrests-chair at a distance of approximately 70–90 cm in front of the computer monitor for presenting stimuli. Four tasks composed the whole experimental procedure, including demographic variable collecting, priming task, emotional words judgment task and debrief.

**Fig 1:**
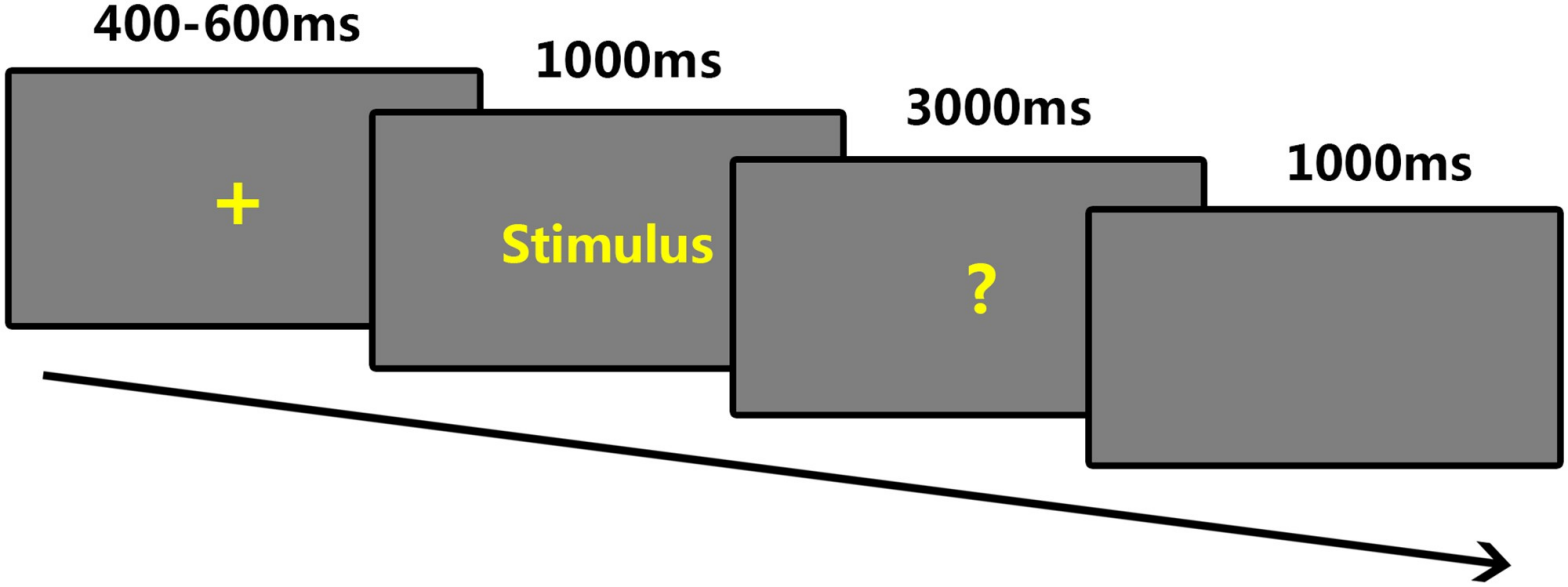
Illustration of the experimental procedure in emotional words judgment task.

Firstly, participants’ demographic information was collected. Secondly, the participants were allocated to the MS priming or control priming randomly by means of finishing the MS task or control task, answering questions on paper. The emotional words judgment task took place in a soundproof, dimly lit room, presented through the E-Prime 2.0 software (Psychology Software Tools, Inc). Fig 1 shows the stimuli presentation sequence. Each trial began with a fixation period of 400 to 600ms. Then a word appeared and remained on the screen of the duration of 1,000ms (Fig 1). Presented in yellow 20 pt Microsoft YaHei Font, experimental words were set on a gray background and each stimulus presented four times within each participant. All experimental words were pseudo randomized that words with the same Word category would not present successively more than three time. In a subsequent response interface, participants were given 3,000ms to judge if the presented stimulus was an emotional word or not and make a prompt response by pressing the corresponding key on the keyboard. Preceded by the fixation of the next trial, the screen was blank for 1,000ms. Ten extra words were used in the practice stage which would be not in the formal experiment. Each word was presented 4 times, in total, 204 words (68 examples of each word category) were shown during the formal task. Stimuli presentation paused automatically after 102 stimuli and participants were able to take a rest which keeps them from getting fatigue, and continued the experiment when they were ready. The experiment lasted 35 mins overall. Accuracy, reaction time and EEG responses to each item were recorded.

### ERP signal recording

The electroencephalogram (EEG) signals were recorded and analyzed using the Neuroscan Synamps system (Neuroscan, USA) which equipped with a 64-channel elastic cap (Electro-Cap International). Distribution of the electrodes complied with the international 10–20 system standard. EEG was amplified with Synamps2 amplifiers (Neuroscan Compumedics) with an AC recording and was sampled continuously at a rate of 1,000Hz. The electrode located halfway between CZ and CPZ electrodes were set as the online reference. The electrode GND was set as grounding on the cap. During the whole experiment, bipolar horizontal electrooculography was recorded by two electrodes setting 1cm outside of the left and right eyes and vertical electrooculography was recorded by two electrodes setting 1.5cm above and below the left eye for artifact rejection purpose. In the process of experiment, electrode impedances were kept below 5kΩ. The participants were asked to hold still and avoid significant eye movement as possible as they could during the experiment.

### Data analysis

EEG data was analyzed by Scan 4.5 software (Neuroscan Compumedics), while SPSS Statistics 22.0 software was used to make succedent statistical analysis of ERPs and behavioral results. Gpower 3 software [39] was applied to measure the statistical power and effect Size.

First, the EEG data were re-referenced off-line to a computed average signal of the referenced left and right mastoid electrodes. Then, Ocular artifact reduction was used to reduce the impact of EOG [40]. After correcting eye movement, 1100ms individual epoches covering the 200ms before and the 900ms after the stimulus onset was picked up from the continuous EEG recordings. The 200ms pre-stimulus interval was used for baseline correction. In addition, further analysis deleted epoches with an amplitude greater than ± 100μV, or contaminated by eye-blinking, other noise factors [41] or with incorrect recognition judgments [42]. After that, the EEG was digitally low-pass filtered at 30Hz (24 db/oct, zero phase shift, Butterworth). ERPs were produced by stimulus-related averaging of the EEG recorded in each condition.

Based on both existing literature [43–46] and visual inspection of the grand average ERPs waveforms, measurement channels were selected and two electrode pools were formed. Based on previous literature [47–49], time-windows for each electrode pools were chosen. The mean P1 amplitude was measured using the average data at the electrode sites of P7, P8, PO5, PO6 within the interval of 100-150ms. The mean amplitude of the N400 was measured at the electrode sites of C3, C1, CZ, C2, C4, CP3, CP1, CPZ, CP2, CP4, P1, PZ, P2 within the interval of 280-460ms.

One participant that completed the experimental task using the same responses to all stimuli was excluded from further behavioral and ERPs data analysis. Seven participants data were exempted because of their excessive EEG artefacts when rejecting artifact (i.e., EEG data drifted seriously) or their poor EEG recording quality (i.e., the ERPs in any condition was averaged less than 30 time). Therefore, a final sample of 53 participants was retained for the behavioral and ERPs analysis.

The trial which beyond three standard deviations from the mean reaction time was excluded. Only reaction time for words that were correctly judged were analyzed. As a result, 63.58 (*SD* = 3.21) trials of anxiety words (MS group: *M* = 63.22 *SD* = 3.18; control group: *M* = 63.96 *SD* = 3.27), 61.85 (*SD* = 5.75) trials of fear words (MS group: *M* = 61.8, *SD* = 5.91; control group: *M* = 61.88 *SD* = 5.70) and 62.64 (*SD* = 4.31) trials of neutral words (MS group: *M* = 62.30, *SD* = 4.31; control group: *M* = 63.00, *SD* = 5.06) were left. We conducted a 2 ×3 mixed repeated-measure ANOVA with between-subject variable of Group (MS group, control group) and a within-subject variable of Word category (anxiety, fear, neutral) was applied to measure the number of trials. We did not find any main effect or other interaction effects of the number of trials (*ps* > 0.05).

A 2 × 3 mixed repeated-measure ANOVA with a between-subject variable of Group (MS group, control group) and a within-subject variable of Word category (anxiety, fear, neutral) was applied to measure the behavioral performances and ERPs components.

To exclude that results were driven largely by female participants (MS group: 16 females and 11 males; control group: 16 females and 10 males), a 2 × 2 × 3 mixed repeated-measure ANOVA with between-subject variable of Group (MS group, control group) and Gender (male, female), a within-subject variable of Word category (anxiety, fear, neutral) was applied to measure the behavioral performances (accuracies and reaction time) and ERPs components. All significant effects and p-values were corrected for non-sphericity by using Greenhouse–Geisser corrections. Bonferroni correction was used to investigate significant main and interaction effects and account for multiple post-hoc comparisons.

## Results

### Behavioral results

#### Effects of MS on accuracies

Behavioral data are shown in Table 1. Accuracies of all participants were high (94.81%).

**Table 1:** Behavioral results in emotional words judgment task. (mean ± SD).

	Group	N	Word category		
			anxiety	fear	neutral
Accuracy (%)	MS	27	.96 ±.04	.93 ±.08	.95 ±.05
	Control	26	.96 ±.04	.93 ±.08	.95 ±.07
Reaction Time (ms)	MS	27	355.71 ± 114.45	367.31 ± 113.13	336.30 ± 108.34
	Control	26	424.68 ± 98.40	424.50 ± 90.26	362.96 ± 81.23

ANOVA results of accuracies are shown in Table 2. The 2(Group) × 3(Word category) mixed repeated-measures ANOVA of accuracies showed no significant main effect of Group (*F* (1,51) = .03, *p* = .87, *1-β* = .05, *f* = .01, *η*^*2*^_*p*_ = .0002). The main effect of Word category was not found (*F* (2,102) = 2.88, *p* = .07, *1-β* = .97, *f* = .24, *η*^*2*^_*p*_ = .05). And the Group × Word category interaction was not found (*F* (2,102) = .03, *p* = .97, *1-β* = .11, *f* = .05, *η*^*2*^_*p*_ = .002). These results suggested that MS manipulation and word categories did not affect participants’ access to meaning of words.

**Table 2:** Mixed repeated-measures ANOVA of accuracy.

	df	F	*p*	*1-β*	*f*	*η*^*2*^_*p*_
Group	1	.03	.87	.05	.01	.0002
Word category	2	2.88	.07	.97	.24	.05
Group × Word category	2	.03	.97	.11	.05	.002

#### Effects of MS on reaction time

ANOVA results of reaction time are shown in Table 3. The 2(Group) × 3(Word category) mixed repeated-measures ANOVA of mean reaction time showed a significant main effect of Group (*F* (1,51) = 4.88, *p* = .03, *1-β* = 1.00, *f* = 1.29, *η*^*2*^_*p*_ = .63), as mean reaction time of words was longer in control group than MS group. The main effect of Word category was also significant (*F* (2,102) = 18.52, *p* < .001, *1-β* = 1.00, *f* = .61, *η*^*2*^_*p*_ = .27). Bonferroni corrected post-hoc comparisons revealed that reaction time to neutral words was significantly shorter than anxiety words (*p* < .001) and fear words (*p* < .001), but reaction time to anxiety and fear words did not differ from each other (*p* = .63). Interaction of Group and Word category was not significant (*F* (2,102) = .95, *p* = .39, *1-β* = .56, *f* = .14, *η*^*2*^_*p*_ = .02). These results suggested that reaction time to words in control group were longer than MS group. Moreover, reaction time of neutral words was shorter than anxiety and fear words.

**Table 3:** Mixed repeated-measures ANOVA of reaction time.

	df	F	*p*	*1-β*	*f*	*η*^*2*^_*p*_
Group	1	4.88	.03*	1.00	1.29	.63
Word category	2	18.52	< .001**	1.00	.61	.27
Gender × Word category	2	.95	.39	.56	.14	.02

#### Effects of gender on accuracy and reaction time

Statistical results are shown in Table 4. The 2(Group) × 2(Gender) × 3(Word category) mixed repeated-measure ANOVA of accuracy showed no significant main effect of Group (*p* = .90), Gender (*p* = .96) and Word category (*p* = .07). And the interaction of Group × Gender (*p* = .89), Group × Word category (*p* = .95), Gender × Word category (*p* = .84), Group × Gender × Word category (*p* = .92) was not found. These results suggested that gender did not affect participants’ access to meaning of words.

**Table 4:** Mixed repeated-measures ANOVA of accuracies and reaction time.

		df	F	*p*	*1-β*	*f*	*η*^*2*^_*p*_
Accuracy	Group	1	.02	.90	.11	.18	< .001
	Gender	1	.002	.96	.13	.21	< .001
	Word category	2	2.79	.07	1.00	.24	.05
	Group × Gender	1	.02	.89	.05	.02	< .001
	Group × Word category	2	.05	.95	.07	.06	.001
	Gender × Word category	2	.17	.84	.07	.07	.003
	Group × Gender ×Word category	2	.09	.92	.06	.05	.002
Reaction Time	Group	1	.50	.49	.16	.25	.01
	Gender	1	3.03	.09	.07	.10	.06
	Word category	2	17.78	< .001**	.99	.60	.27
	Group × Gender	1	3.04	.09	.16	.25	.06
	Group × Word category	2	1.67	.19	.24	.18	.03
	Gender × Word category	2	1.08	.34	.17	.15	.02
	Group × Gender × Word category	2	.22	.80	.07	.07	.004

The 2(Group) × 2(Gender) × 3(Word category) mixed repeated-measure ANOVA of reaction time showed no significant main effect of Group (*p* = .90) and Gender (*p* = .96). But the main effect of Word category (*p* = .07) was significant, as reaction time to neutral words was significantly shorter than anxiety words (*p* = .001) and fear words (*p* < .001), but reaction time to anxiety and fear words did not differ from each other (*p* = .15). Interaction of Group × Gender (*p* = .09), Group × Word category (*p* = .19), Gender × Word category (*p* = .34), Group × Gender × Word category (*p* = .80) was not found.

### ERPs results

#### Waveforms description

**Fig 2:**
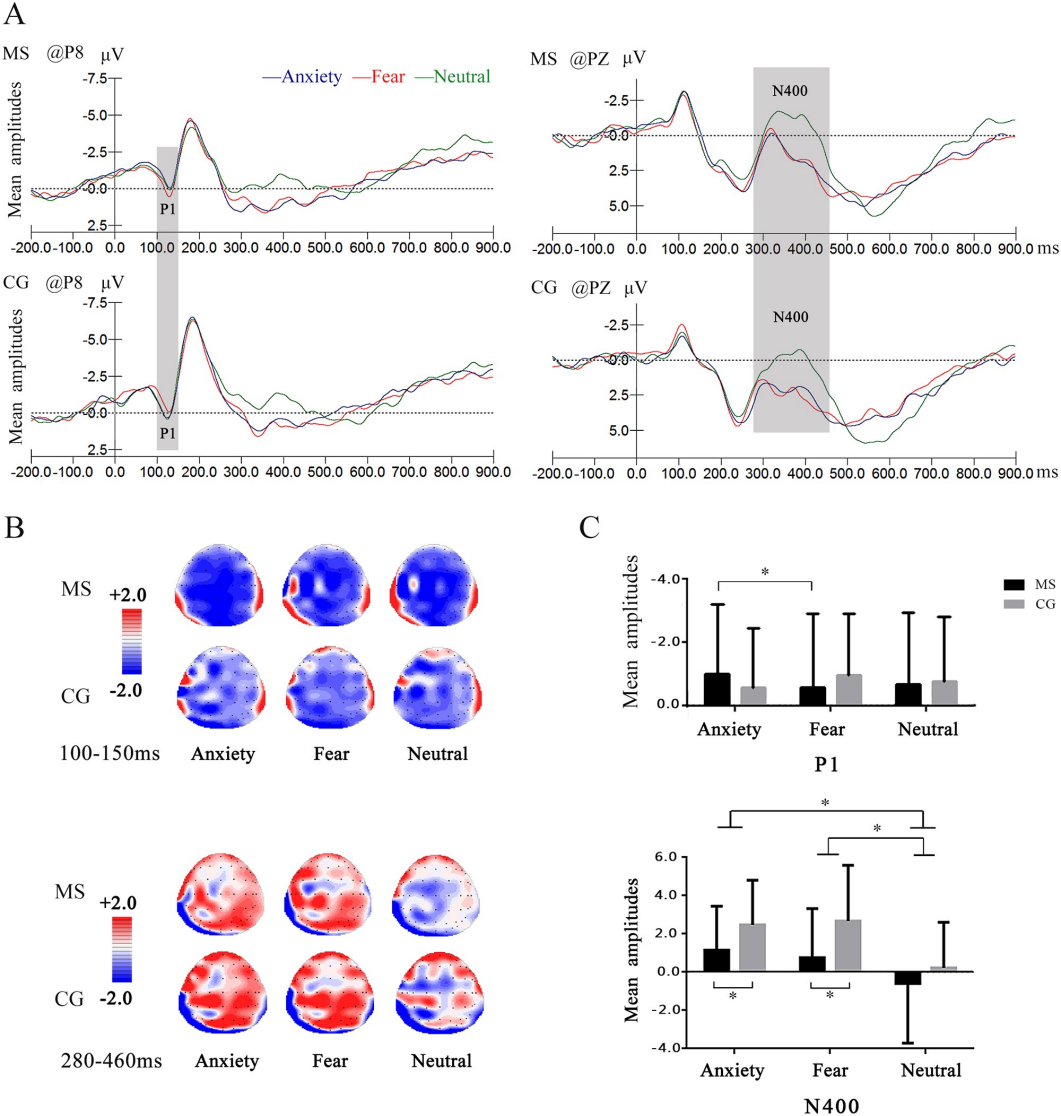
The results of ERPs. (A) Results for main effects of emotion in the P1 and N400. MS = Mortality Salience group, CG = control group. Grand average ERP waveforms for each type of words in two groups at P8 and PZ. (B) The corresponding scalp topographies of the P1 and N400 component, P1 is illustrated on the top while N400 on the below. (C) Bar graphs of the average amplitude of P1 and N400. Black and gray bars represent the MS group and control group, respectively. Error bars represent standard deviation and *p < .05.

As is shown in Fig 2A, we could see the waveform elicited by the words. There was a P1 locating in occipital and a negative deflection during 280 ms– 460 ms which could be regarded as N400. Based on visual inspection, condition differences emerged mainly at P1 and N400. The following analyses were performed particularly focusing on P1 and N400.

#### Descriptive statistics

Table 5 describes the descriptive statistics of the ERPs for the MS group and the control group of P1and N400 amplitudes. Grand-averaged ERPs elicited by MS-Anxiety, MS-Fear, MS-Neutral, control-Anxiety, control-Fear and control-Neutral are illustrated in Fig 2B and 2C. The mean amplitudes of P1 and N400 are depicted in Fig 2C.

**Table 5:** Means, standard deviations among ERPs amplitudes for the MS group and the control group. (mean ± SD).

	Group	N	Word category		
			Anxiety	Fear	Neutral
P1	MS	27	-.99 ± 2.17	-.55 ± 2.32	-.68 ± 2.23
	Control	26	-.56 ± 1.85	-.95 ± 1.93	-.77 ± 2.01
N400	MS	27	1.15 ± 2.30	.77 ± 2.56	-.58 ± 3.13
	Control	26	2.48 ± 2.34	2.68 ± 2.92	.23 ± 2.39

#### P1

ANOVA results of P1 are shown in Table 6. The 2(Group) × 3(Word category) mixed repeated-measures ANOVA of the mean P1 amplitudes within the interval of 100-150ms showed nonsignificant main effects of Group (*F* (1,51) = .002, *p* = .97, *1-β* = .05, *f* = .005, *η*^*2*^_*p*_ = .002) and Word category (*F* (2,102) = .05, *p* = .95, *1-β* = .08, *f* = .03, *η*^*2*^_*p*_ = .001). However, the interaction of Group × Word category was significant (*F* (2,102) = 3.53, *p* = .04, *1-β* = .99, *f* = .26, *η*^*2*^_*p*_ = .07). Simple effect tests showed that the mean P1 amplitudes of MS group elicited by fear words were more positive than anxiety words (*p* = .04), but neutral words did not differ from anxiety words (*p* = .56) and fear words (*p* = 1.00). These effects were not found in the control group that P1 amplitudes elicited by anxiety, fear and neutral words did not different from each other.

**Table 6:** Mixed repeated-measures ANOVA of P1.

	df	F	*p*	*1-β*	*f*	*η*^*2*^_*p*_
Group	1	.002	.97	.05	.005	.002
Word category	2	.05	.95	.08	.03	.001
Group × Word category	2	3.53	.04*	.99	.26	.07

#### N400

ANOVA results of N400 are shown in Table 7. The 2(Group) × 3(Word category) mixed repeated-measures ANOVA of the mean N400 amplitudes within the interval of 280-460ms showed a significant main effect of Group (*F* (1,51) = 4.34, *p* = .04, *1-β* = .72, *f* = .29, *η*^*2*^_*p*_ = .08), as MS group elicited a more negative N400 amplitude than control group. Bonferroni corrected post-hoc comparisons revealed that mean N400 amplitudes of anxiety words and fear words in the MS group were more negative than that of control group (*ps* < 0.05), whereas no difference in neutral words (*p* = .29) between these two groups. A significant main effect of Word category was observed (*F* (2,102) = 33.28, *p* < .001, *1-β* = 1.00, *f* = .81, *η*^*2*^_*p*_ = .39), as neutral words elicited larger amplitudes than anxiety words (*p* < .001) and fear words (*p* < .001), whereas no difference between anxiety words and fear words (*p* = .73). There was no significant interaction between Group and Word category (*F* (2,102) = 1.95, *p* = .15, *1-β* = .88, *f* = .20, *η*^*2*^_*p*_ = .04).

**Table 7:** Mixed repeated-measures ANOVA of N400.

	df	F	*p*	*1-β*	*f*	*η*^*2*^_*p*_
Group	1	4.34	.04*	.72	.29	.08
Word category	2	33.28	< .001**	1.00	.81	.39
Group × Word category	2	1.95	.15	.88	.20	.04

#### Effects of gender on ERPs

Statistical results are shown in Table 8. The 2(Group) × 2(Gender) × 3(Word category) mixed repeated-measure ANOVA of P1 showed no significant main effect of Group (*p* = .90), Gender (*p* = .79) and Word category (*p* = .99). Interaction of Group × Word category (*p* = .03) was significant. Simple effect tests showed that the mean P1 amplitudes of MS group elicited by fear words were more positive than anxiety words (p = .05), but neutral words did not differ from anxiety words (p = .07) and fear words (p = 1.00). These effects were not found in the control group. But the interaction of Group × Gender (*p* = .70), Gender × Word category (*p* = .06), Group × Gender × Word category (*p* = .78) was not found.

**Table 8:** Mixed repeated-measures ANOVA of P1 and N400.

		df	F	*p*	*1-β*	*f*	*η*^*2*^_*p*_
P1	Group	1	.02	.90	.05	.02	< .001
	Gender	1	.07	.79	.05	.04	.001
	Word category	2	.01	.99	.05	.02	< .001
	Group × Gender	1	.15	.70	.06	.06	.003
	Group × Word category	2	3.54	.03*	.46	.27	.07
	Gender × Word category	2	2.82	.06	.38	.24	.05
	Group ×Gender ×Word category	2	.25	.78	.07	.07	.01
N400	Group	1	4.68	.04*	.22	.31	.09
	Gender	1	3.18	.08	.17	.25	.06
	Word category	2	30.64	< .001**	1.00	.79	.39
	Group × Gender	1	.09	.77	.04	.06	.002
	Group × Word category	2	1.70	.19	.24	.19	.03
	Gender × Word category	2	.04	.96	.05	.03	.001
	Group ×Gender ×Word category	2	.04	.97	.03	.03	.001

The 2(Group) × 2(Gender) × 3(Word category) mixed repeated-measure ANOVA of N400 showed significant main effect of Group (*p* = .04), as MS group elicited a more negative N400 amplitude than control group. A significant main effect of Word category was observed (*p* < .001), as neutral words elicited larger amplitudes than anxiety words (*p* < .001) and fear words (*p* < .001), whereas no difference between anxiety words and fear words (*p* = 1.00). Nonsignificant main effect was found of Gender (*p* = .08). Interaction of Group × Gender (*p* = .77), Group × Word category (*p* = .19), Gender × Word category (*p* = .96), Group × Gender × Word category (*p* = .97) was not found.

#### Summary of the ERP findings

We found that fear words elicited a larger P1 than anxiety words in MS group, but not in control group. A main effect of group on N400 indicated that MS group showed a larger N400 compared to control group. Moreover, smaller N400 was found in anxiety and fear words than neutral words, while no difference was found between fear and anxiety words.

## Discussion

The present EEG study aimed to advance the extant literature by investigating emotional components and corresponding neural reactions in terror management process. Behavioral results showed that reaction time in control group was longer than MS group. A shorter reaction time was showed in neutral words than anxiety and fear words. ERPs results showed that a more positive P1 was elicited by fear words than anxiety words after MS, but we did not find this effect in control group. In addition, N400 amplitudes were more negative in MS group compared to control group. Meanwhile, neutral words elicited a larger amplitude of N400 than anxiety words and fear words, whereas no difference between anxiety words and fear words was found.

The present study showed that fear and anxiety words’ reaction time were longer than neutral words. When it comes to reaction time, a negative delay effect that individuals react slower to negative words than to positive and neutral words was found on research of lexical decision [50] and lexical naming [51], notes that it takes longer to disengage from negative stimuli. Threatening information has important biological significance, which makes the individuals stick on the stimulus for a longer time and become difficult to divert attention [52]. Fear words and anxiety words are threatening stimuli for individuals which might elicit attention disengagement. In addition, assuming that the proximal defense is accompanied by emotional responses, the individuals will inhibit fear and anxiety words, rather than neutral words. However, MS group had shorter reaction times than control group. This result shows that after mortality salience, individuals’ judgment of words become faster throughout the task. Generally, there are two types of control groups in the mortality salience experiment. One is the negative/threat but death-unrelated control group (e.g., think about toothache, body pain, or subliminal expressions of "pain"), which are suitable to distinguish the different behavioral responses caused by death information and other negative thoughts, so as to highlight the special influence of death thoughts on individuals. The other is the neutral/active but death-unrelated control group (e.g., think about daily non-death neutral topics or subliminal presentation of "neutral" words), since it does not cause emotion-related content, it is suitable for comparing the generation of emotion after mortality salience. It is worth noting that the control group in this study is the neutral group rather than the toothache group. Difference of reaction time between the groups was not caused by negative emotions of stimulus but death-related emotions, reflecting more cognitive avoidance during the proximal defense process of terror management. But the behavioral results are not yet sufficient to support the suppression of negative emotions during the proximal defense.

P1 reflects early attentional modulation on the conscious level [53, 54]. A larger wave of P1 indicates the allocation of attention resources [21]. Our results of P1 revealed that fear words elicited a larger P1 than anxiety words in MS group, while this effect was not significant in control group. The distinct effects between fear and anxiety words may suggest that fear words receive more attention than anxiety words after MS, which suggests that terror management process induce fear emotion.

Regarding N400 component, neutral words induced larger N400 than the fear and anxiety words. ERP studies on words processing revealed that difficulty in semantic understanding elicited larger N400 amplitude, N400 was distinctly sensitive to the processing of lexical access [55]. In this experiment, individuals were required to judge the emotional meaning of neutral words and negative words among the three types of words. Since the three types of words had the same number, one-third of the trials were for neutral words, and two-thirds of the trials were for negative words. Neutral words were less likely to appear than the other two emotional words. Neutral words were more unexpected and difficult for the participants, which showed larger N400 amplitude. Compared with control group, MS group induced a larger N400. N400 is a component associated with the difficulty in integrating [56] and identifying words [57]. Specifically, the violation of the context of semantic priming was indicated to be associated with the N400 component, a more negative-going wave was elicited by the unrelated emotional target than those related [58–60]. Enhanced negative deflection might reflect larger cognitive inhibition of words processing and more cognition effort [27, 61]. Larger N400 in MS group indicated that individuals were more difficult to gain access to words than the control group. Although the statistical explanation of the results that the mean N400 amplitudes of anxiety words and fear words in the MS group were more negative than that of control group are not yet sufficient, such patterns are reflected in the descriptive results. Results of N400 supports the proximal defense hypothesis of terror management theory, that is, more cognition resources are needed to suppress death-related emotions, thus affecting the accessibility of words in the current study.

Given the imbalance of male/female participants, gender was taken into account in the ANOVA as a between-subjects factor to exclude that results are driven largely by female participants only. The results of behavioral and neural responds did not find the influence of gender. But there is no consensus on the relationship between gender and death-related emotions at present [62–64]. For further research, it’s necessary to investigate the influence of gender on fear and anxiety after exposure to death information.

The current research has several limitations. First, traditional reaction time measurement does not provide real-time measurement due to delay caused by button reactions, limiting the direct comparison of emotional processing distinction. In order to obtain more real-time physiological information, other electrophysiological methods such as Skin conductance responses (SCR) [65] and autonomic system activity measure [66] can be exploited in the future experiment. Due to the technical limitation of ERPs, the low spatial resolution of ERPs cannot provide confirmatory evidence on the neuroanatomical generator of an ERP component and indicate the deep located structures within the brain [67, 68]. Further study can integrate functional magnetic resonance imaging (fMRI) method with ERPs to identify the emotion processing patterns of brain activation caused by death-related thoughts. Second, Since the three types of words (neutral, fear, anxiety) had the same number, neutral words were less likely to appear than the other two emotional words. In our experiment, we paid more attention to the participants’ different response patterns to emotional words. When it comes to P1, nonsignificant difference of neutral words between MS and control group and no significant differences between neutral words and emotion words within each group were found. In other words, the patterns of P1 of neutral words were relatively consistent. Descriptive results reflect that the patterns of N400 were similar of neutral words between these two groups. Based on the above results, we believe that the unbalanced number of words did not affect the ERPs finding. In further experiments, neutral words should be added to balance the number of neutral and emotional words. Third, the participants taken part in this study are quite young, whether the elderly and young people have the same pattern in the face of death-related thoughts is still unclear. A growing body of research suggested that there are age-related differences in the face of death information. Increasing age brings older adults’ increasing closeness to life’s end. Older adults experience increasingly frequent reminders of mortality due to their own susceptibility to physical ailments, as well as the loss of loved ones. Some research showed that older adults focused more on positive information and used more positive strategies, while attending less to negative information [69–71]. The empirical research found that the subjective experience of negative emotions such as anger and fear in the elderly is significantly lower than that in the young [63, 72–78]. Studies have shown that the acceptance of death increased with age [79, 80], which reflected the higher emotional control of older adults and their more flexible respond to reminders of mortality [81]. It is worthwhile to study the patterns of older people in the face of death information, which are related to the quality of life and might complement the terror management theory.

## Conclusion

The current research shows that death-related thought will induce fear and anxiety in the early stage, but soon these negative emotions are suppressed and are at a lower level of accessibility. The findings provide evidence that terror management process involves not only cognitive components, but also emotional components during proximal defense, which helps to enrich terror management theory.

## Supporting information

S1 Data (XLSX)
